# Enhanced expression of the soluble form of E-selectin attenuates progression of lupus nephritis and vasculitis in MRL/lpr mice

**DOI:** 10.1002/iid3.6

**Published:** 2013-10-30

**Authors:** Kimihiko Nakatani, Shuhei Yoshimoto, Osamu Asai, Hirokazu Sakan, Miho Terada, Yoshihiko Saito, Masato Nose, Masayuki Iwano, Noboru Konishi

**Affiliations:** 1 Department of Pathology, Nara Medical UniversityKashihara, Nara, Japan; 2 First Department of Internal Medicine, Nara Medical UniversityKashihara, Nara, Japan; 3 Division of Pathogenomics, Department of Pathology, Ehime University Graduate School of MedicineTo-on, Ehime, Japan; 4 Division of Nephrology, Department of General Medicine, Faculty of Medical Sciences, University of FukuiYoshida-gun, Fukui, Japan

**Keywords:** Lupus, MRL/lpr mice, nephritis, soluble E-selectin, vasculitis

## Abstract

Systemic lupus erythematosus (SLE) is a chronic autoimmune disease that causes inflammatory tissue damage, including lupus nephritis and vasculitis. Local generation of adhesion molecules and expression of their ligands on inflammatory cells appears to contribute to the progression of SLE. We found significantly increased E-selectin expression in the glomeruli and renal interstitial microvasculature of MRL/MpJ-*lpr/lpr* (MRL/lpr) lupus model mice. This was accompanied with infiltration of inflammatory cells, especially macrophages and CD8^+^ T cells. Similarly, in 21 patients with proliferative lupus nephritis, there was a significant correlation between renal E-selectin levels and macrophage and CD8^+^ T cell infiltration in the affected kidneys. By contrast, in transgenic MRL/lpr mice exhibiting elevated levels of circulating soluble E-selectin (sE-selectin) protein, which competitively inhibits E- and P-selectin-mediated extravasation of inflammatory cells, the progression of lupus nephritis and vasculitis was significantly suppressed and survival was significantly prolonged. This improvement was accompanied by significant reductions in renal infiltration by macrophages and CD8^+^ T cells. These results suggest that E-selectin plays a crucial role in lupus nephritis and vasculitis by mediating renal infiltration of inflammatory cells, and that because it inhibits this process, sE-selectin could potentially serve as an effective treatment for lupus nephritis and vasculitis.

## Introduction

Systemic lupus erythematosus (SLE) is an autoimmune disorder characterised by polyclonal expansion of autoreactive lymphocytes, production of multiple autoantibodies, immune complex (IC) deposition in the microvasculature of various organs, complement activation, leukocyte infiltration and tissue damage [1]. IC-mediated glomerulonephritis and renal vasculitis are major causes of morbidity in SLE and are key determinants of disease outcome [2]. In lupus nephritis and vasculitis, ICs trigger the synthesis of various inflammatory mediators, resulting in cellular infiltration and progressive renal damage [3,4]. In response to IC deposition, renal endothelial cells locally express adhesion molecules, which in turn mediate leukocyte recruitment during the initiation and amplification phases of renal inflammation [3,4]. The selectin family of adhesion molecules (e.g., E- and P-selectin) and their carbohydrate-containing ligands (e.g., sialyl-Lewisx or sialyl-Lewisa) are expressed on activated vascular endothelial cells and circulating leukocytes, respectively, and the resultant selectin-mediated cell–cell interactions are prerequisites for subsequent firm attachment and transmigration of leukocytes [5–8]. Indeed, we previously reported the contribution made by E-selectin to the progression of endocapillary proliferative glomerular lesions caused by macrophage infiltration in an experimental lupus model mouse [9].

Renal infiltration of effector CD8^+^ T cells expressing various chemokine receptors and adhesion molecule ligands, which facilitate recruitment into peripheral tissues, especially at sites of inflammation, also play a critical role in the development and progression of lupus nephritis [10–12]. One of the adhesion molecule ligands expressed by effector CD8^+^ T cells is a functional glycoprotein that can bind to E- and P-selectin on endothelial cells [13]. However, it remains controversial as to whether E-selectin expression contributes to the aggregation of CD8^+^T cells in kidneys affected by lupus nephritis and vasculitis.

In this present study, we first demonstrated that E-selectin expression in glomeruli and small vessels gradually increases during the development of lupus nephritis and vasculitis in *MRL/MpJ-lpr/lpr* (MRL/lpr) lupus mice, and that this is accompanied by significant infiltration of the glomeruli and perivascular regions by CD68^+^ macrophages, CD4^+^ T cells and CD8^+^ T cells. We also showed that in human proliferative lupus nephritis, levels of renal E-selectin expression are significantly associated with renal infiltration by both CD8^+^ T cells and CD68^+^ macrophages, but not by CD4^+^ T cells. Finally, we showed that high serum levels of soluble E-selectin protein (sE-selectin) ameliorates the progression of lupus nephritis and vasculitis by suppressing infiltration of both CD8^+^ T cells and CD68^+^ macrophages in transgenic (Tg) MRL/lpr mice expressing a sE-selectin gene. We therefore propose that sE-selectin exerts a therapeutic effect on lupus nephritis and vasculitis through abrogation of CD8^+^ T cell and macrophage migration.

## Materials and Methods

### Animals

MRL/MpJ-Faslpr (MRL/lpr) mice were purchased from The Jackson Laboratory and maintained as a breeding colony in our animal facility. Transgenic mice harboring the sE-selectin genes were established as previously described [14]. In brief, to construct the cDNA encoding the soluble form of E-selectin, PCR was used to introduce a stop codon just before the transmembrane region of E-selectin cDNA. Soluble E-selectin cDNA was then cloned into an expression vector also containing the human α-1-antitrypsin promoter (AAT) and a portion of the rabbit β-globin gene, so that the human AAT promoter drove expression of the resultant chimeric gene selectively in the liver. The transgene was then microinjected into fertilised eggs obtained from C57BL/6xDBA2 F1 mice. These transgenic mice were backcrossed for at least 12 generations into the MRL/lpr background to obtain sE-selectin Tg MRL/lpr animals. Genomic DNA from sE-selectin Tg mice backcrossed to MRL/lpr was screened for Tg positivity and lpr homozygosity in part using PCR. The primers used to amplify the Tg were 5′-TAC AAG TCC TCA TGT GCC-3′ (forward) and 5′-TTT GAA TTC AGG CCT GGC TGA CTG GGG CTT CAC A-3′ (reverse). The homozygosity of the lpr gene was detected as reported previously [15]. RT-PCR analysis using total RNA from liver of both 20-week-old Tg and wild-type mice showed that the expression of sE-selectin was increased in Tg mice, as compared to wild-type mice (Fig. S4). The following primers were used to detect the E-selectin extracellular domain: 5′-TAC AAG TCC TCA TGT GCC-3′ (forward) and 5′-GGC TTC ACA GGT AGG CGG C -3′ (reverse) (product size: 290 bp). Tg mice backcrossed to MRL/lpr mice more than 12 times were genotyped using a total of 35 polymorphic microsatellite markers, which corresponds to more than one marker per chromosome (Research Genetics, Huntsville, AL, USA), in order to determine the proportion of the genetic composition of MRL and the insertion site of the Tg. Only one marker, at chromosomal position D9Mit229 (chromosome 9, 28.0 cM), was not homozygous for the MRL gene, which indicates the mice used in the present study had a MRL genetic background and that the Tg insertion site was located on chromosome 9. This does not correspond to the map position of nephritis and vasculitis-susceptible loci of MRL/lpr mice [16,17]. All mice were housed under specific pathogen-free conditions. To minimise genetic background differences between the animals, the wild-type MRL/lpr mice used in this study were derived from the same litter as the sE-selectin Tg MRL/lpr animals. Animal experiments were performed according to national and institutional animal care and ethical guidelines, and were approved by local committees.

### Real-time RT-PCR analysis

Total renal RNA was prepared from the cortex of frozen murine kidneys, as described previously [9]. RNA from archived frozen human biopsy specimens was extracted from two 10-µm-thick sections from each patient, as described previously [18]. For real-time PCR, 1 μl of each first-strand reaction product was amplified with appropriate primers and the corresponding fluorescent probes for human and murine E-selectin (assay IDs: Hs00950401_m1, Mm00441278_m1), murine IFNγ (assay IDs: Mm01168134_m1), TNFα (assay IDs: Mm00443258_m1), human β-actin (assay ID: Hs00242273_m1), or murine glyceraldehyde-3-phosphate dehydrogenase (assay ID: Mm00484668_m1). These probes were designed by the Applied Biosystems ‘Assay-on-Demand’ service (Foster City, CA). The human E-selectin/β-actin mRNA, murine E-selectin/glyceraldehyde-3-phosphate dehydrogenase mRNA, murine IFN-γ/glyceraldehyde-3-phosphate dehydrogenase mRNA, and murine TNF-α/glyceraldehydes-3-phosphate dehydrogenase mRNA ratios were calculated for each sample.

### Morphological examination

Tissue samples were fixed with 10% formalin in 0.01 mol/L phosphate buffer (pH 7.2), embedded in paraffin and stained with hematoxylin and eosin (H&E) or periodic acid-Schiff (PAS) for histological examination. The severity of the lesions in each glomerulus in mice was estimated by grading from 0 to 3, where grade 0 is normal, grade 1 has limited segmental mesangial proliferation, grade 2 has endocapillary proliferation with segmental wire loop and/or hyaline thrombotic lesions, and grade 3 has dominant sclerosis and/or hyalinosis of the lesions in grade 2. The glomerular lesion index refers to the average grade in each mouse. Similarly, the severity of renal vasculitis in was estimated in each mouse on a scale from 0 to 3, where grade 0 is normal; grade 1 shows perivascular mononuclear cell infiltration, but no destruction of the external elastic lamina; grade 2 shows marked accumulation of mononuclear cell infiltrates with microgranuloma formation close to the external elastic lamina and destruction of the external elastic lamina; and grade 3 shows intimal thickening in the artery with myointimal cell proliferation in addition to the lesions observed in grade 2. The renal vascular lesion index refers to the average grade in each mouse.

### Immunohistochemistry

Frozen sections (4 µm) of mouse kidney were stained with antibodies directed against a macrophage marker (CD68; BD Pharmingen, San Diego, CA) and T cell markers (CD4 and CD8; BD Pharmingen) and then visualised using a Histofine MAX-PO (Nichirei Biosciences, Inc., Tokyo, Japan). Under light microscopy, CD68^+^, CD4^+^ and CD8^+^ cells were counted in a blinded fashion in 20 glomerular cross-sections per kidney, and in four unit areas around small vessels in the kidney. In each mouse, the average counts per one glomerulus or unit area of CD68^+^, CD4^+^ or CD8^+^ cells were presented. Staining for E-selectin was accomplished using biotinylated rat monoclonal anti-murine E-selectin antibodies (BD Pharmingen) followed by FITC-labeled streptavidin (Dako, Glostrup, Denmark). IgG3 was stained using FITC-conjugated rabbit anti-mouse IgG3 antibodies (ICN ImmunoBiologicals, Lisle, IL, USA).

### Measurement of sE-selectin, IgG3, anti-DNA antibodies, albumin, and blood urea nitrogen (BUN) in murine sera

sE-selectin concentrations in murine sera were determined by ELISA, as a previous report [9,19]. Levels of anti-DNA antibodies were evaluated using standard analytic protocols (reaction of anti-DNA antibodies to calf thymus DNA (Sigma–Aldrich, St. Louis, MO)). Albumin and BUN were measured in murine sera by SRL Inc. (Tokyo, Japan).

### Patients and tissue samples

Renal biopsy samples were obtained from 21 patients, in whom World Health Organisation types III and IV lupus nephritis had been diagnosed. Paraffin tissue sections of renal biopsy specimens (2 µm) were placed in ethyendiaminetetraacetic acid (EDTA) buffer (pH 9.1) and heated in a microwave for 25 min or pre-treated with protease for 10 min. For single staining, the tissue was then incubated with monoclonal mouse anti-human CD68 (Dako), monoclonal mouse anti-human CD4 (Dako) and monoclonal mouse anti-human CD8 (Dako) antibodies. The tissue sections were then developed using Histofine MAX-PO (Nichirei Bioscience, Inc.). CD68^+^, CD4^+^ and CD8^+^ cells were counted in a blinded fashion in 10 glomerular cross-sections and 10 unit areas in tubule-interstitium per kidney and the average counts per one glomerulus or one unit area were presented in each patient. All clinical study protocols were approved by the Nara Medical University Ethics Committee (No. 2002–2009). In all cases, written informed consent was obtained from either the patient or his/her family.

### Statistical analysis

Data are expressed as means ± SD. The differences between two groups were evaluated using Student's *t-*test. The Pearson correlation coefficient was used to assess the relationships between levels of renal E-selectin mRNA expression and inflammatory cell counts in human kidneys with lupus nephritis. The comparisons of survival rates were performed with the Kaplan–Meier method, with differences in the survival curves evaluated with a log-rank sum testing. Values of *P* < 0.05 were considered to be significant.

## Results

### E-selectin expression and inflammatory cell infiltration in kidneys from MRL/lpr mice

MRL/lpr mice exhibited progressive development of renal damage that became noticeable by 12 weeks of age. The renal lesions included enlarged hypercellular glomeruli with increases in the numbers of both resident cells and infiltrating leukocytes, and higher levels of mesangial matrices, which are consistent with human endocapillary proliferative glomerulonephritis. With the progression of the renal damage, vasculitis also developed in the affected kidneys. The renal histologic lesions seen in MRL/lpr mice exhibited progressively increasing severities of proliferative glomerulonephritis, accompanied with increasing infiltrating leukocytes and formations of cellular crescents, so that the mice showed signs of severe renal damage by 20 weeks of age.

**Figure 1:**
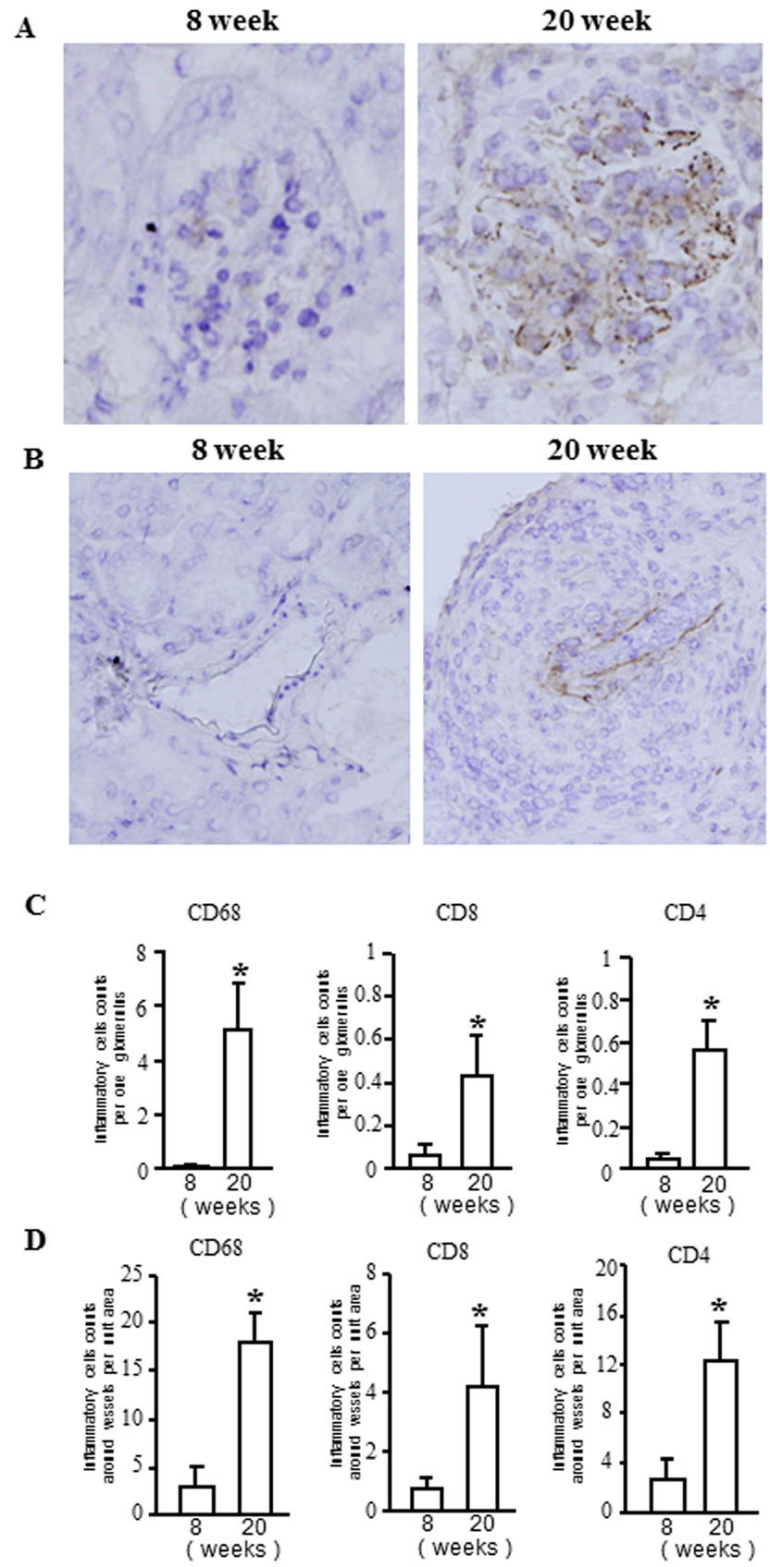
E-selectin expressions and inflammatory cells infiltrations in the kidneys of MRL/lpr mice. (A and B) Immunoperoxidase staining for E-selectin in glomeruli (A) and small vessels (B) in kidneys from 8- and 20-week-old MRL/lpr mice. (C and D) Quantitative analysis of inflammatory cell infiltration of glomeruli (C) and perivascular regions (D) in kidneys from 8- and 20-week-old MRL/lpr mice. Data are expressed as means ± SD. *P < 0.001 versus 8-week-old MRL/lpr mice (*n* = 6 mice per group).

In our first experiment, we used immunohistochemistry to determine the location of E-selectin expression within the kidneys of MRL/lpr mice during the progression of the renal damage (Fig. 1A and B). We found that E-selectin expression was almost undetectable in the glomeruli and tubulointerstitium of 8-week-old MRL/lpr mice, but by 20 weeks significant expression was seen in the glomeruli and, to a lesser extent, in the interstitial microvasculature. E-selectin was predominantly localised along the capillary walls within glomeruli and along the walls of the interstitial microvessels that exhibited vasculitis and were surrounded by inflammatory cells.

In addition, we also found CD68^+^ macrophages, CD4^+^ T cells, and CD8^+^ T cells was accumulated in the glomeruli and perivascular regions of kidneys from 20-week-old MRL/lpr mice (Fig. S1A and B) at levels that were significantly greater than in 8-week-old mice (Fig. 1C and D).

### Association of E-selectin expression with inflammatory cell infiltration in the kidneys of patients with diffuse proliferative lupus nephritis

**Figure 2:**
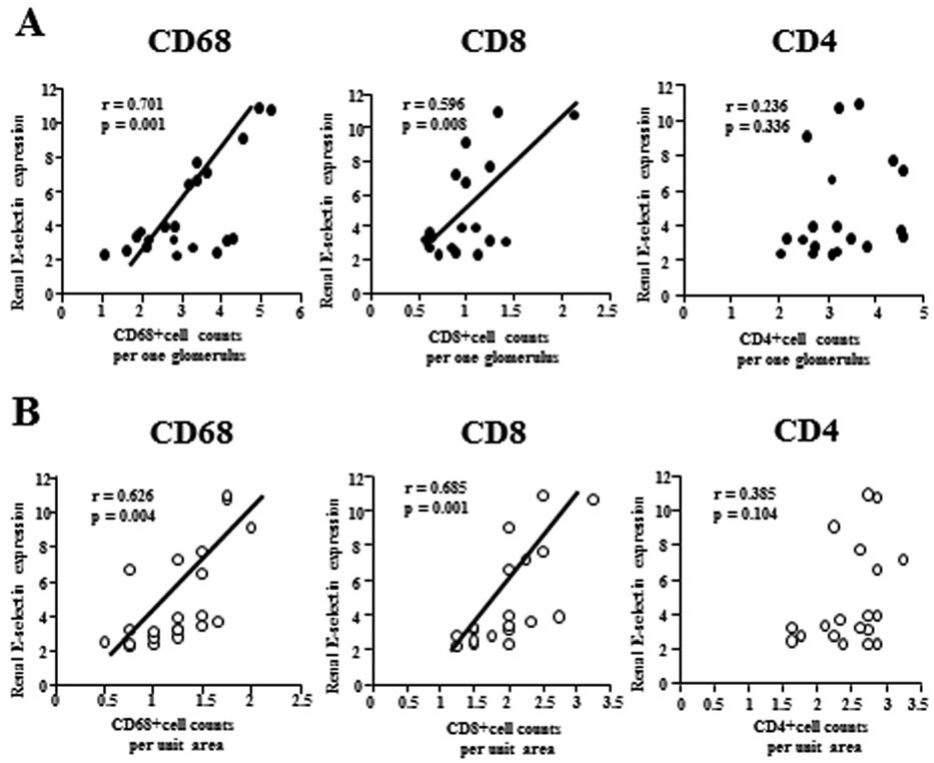
Correlation between levels of E-selectin mRNA expression and infiltration by CD68^+^ macrophages, CD4^+^ T cells and CD8^+^ T cells in renal biopsy specimens from human proliferative lupus nephritis patients. (A and B) Correlation between levels of renal E-selectin mRNA expression and CD68^+^ macrophage counts (glomeruli: *r* = 0.701, *P* = 0.001; interstitium: *r* = 0.626, *P* = 0.004), CD8^+^ T cell counts (glomeruli: *r* = 0.596, *P* = 0.008; interstitium: *r* = 0.685, *P* = 0.001), and CD4^+^ T cell counts (glomeruli: *r* = 0.236, *P* = 0.336; interstitium; *r* = 0.385, *P* = 0.104) in glomeruli (A) and interstitium (B) in renal biopsy specimens from patients with proliferative lupus nephritis. Renal E-selectin mRNA expressions were analysed using real-time PCR. Correlations were evaluated using the Pearson correlation coefficient.

We also investigated whether renal E-selectin expression was related to the glomerular and interstitial infiltration of CD68^+^ macrophages and/or CD4^+^ and CD8^+^ T cells seen in human patients with diffuse proliferative lupus nephritis. Using renal biopsy specimens, we detected E-selectin expression along the capillary walls within glomeruli, and the accumulation of CD68^+^ macrophages, CD4^+^ T cells and CD8^+^ T cells within glomeruli (Fig. S2). In addition, we found significant correlations between renal mRNA levels of E-selectin expression and CD68^+^ macrophage and CD8^+^ T cell counts in the glomeruli (CD68: *r* = 0.701, *P* = 0.001; CD8: *r* = 0.596, *P* = 0.008) and interstitium (CD68: *r* = 0.626, *P* = 0.004; CD8: *r* = 0.685, *P* = 0.001; Fig. 2A and B). However, there was no significant correlation between renal E-selectin expression and glomerular (*r* = 0.236, *P* = 0.336) or interstitial (*r* = 0.385, *P* = 0.104) CD4^+^ T cell counts (Fig. 2A and B).

### Generation of sE-selectin Tg MRL/lpr mice

To further assess the importance of E-selectin in the progression of lupus nephritis and vasculitis, we generated Tg MRL/lpr mice that overexpressed sE-selectin, leading to elevated levels of the protein in the circulation. In these Tg mice, serum sE-selectin levels were significantly higher than in wild-type MRL/lpr mice at 8 and 20 weeks of age (Table1). To determine whether sE-selectin altered the production of IgG3 antibodies, which are recognised to be nephritogenic, or anti-DNA antibodies in MRL/lpr mice, we compared the serum levels of the antibodies in wild-type and sE-selectin Tg MRL/lpr mice. As shown in Table1, there were no significant differences in either of these factors between wild-type and sE-selectin Tg MRL/lpr mice. Thus excess serum sE-selectin does not affect the production of either IgG3 or anti-DNA antibodies in MRL/lpr mice.

**Table 1:** Serum levels in sE-selectin Tg and wild-type MRL/lpr mice

	8 Weeks		20 Weeks	
		
	sE-selectin Tg	Wild-type	sE-selectin Tg	Wild-type
Soluble E-selectin (ng/ml)	8.02 × 10^3^ ± 1.99 × 10^3^*	2.34 ± 0.64	8.11 × 10^3^ ± 0.90 × 10^3^*	12.74 ± 3.09#
IgG3 (mg/ml)	2.32 ± 0.52	2.62 ± 0.64	32.11 ± 4.21#	31.11 ± 3.95#
Anti-DNA antibody (AU)	0.26 ± 0.06	0.27 ± 0.06	1.03 ± 0.11#	1.11 ± 0.17#

* *P* < 0.001, versus wild-type.

# *P* < 0.05, versus 8 weeks.

### Evaluation of lupus nephritis and vasculitis in sE-selectin Tg MRL/lpr mice

**Figure 3:**
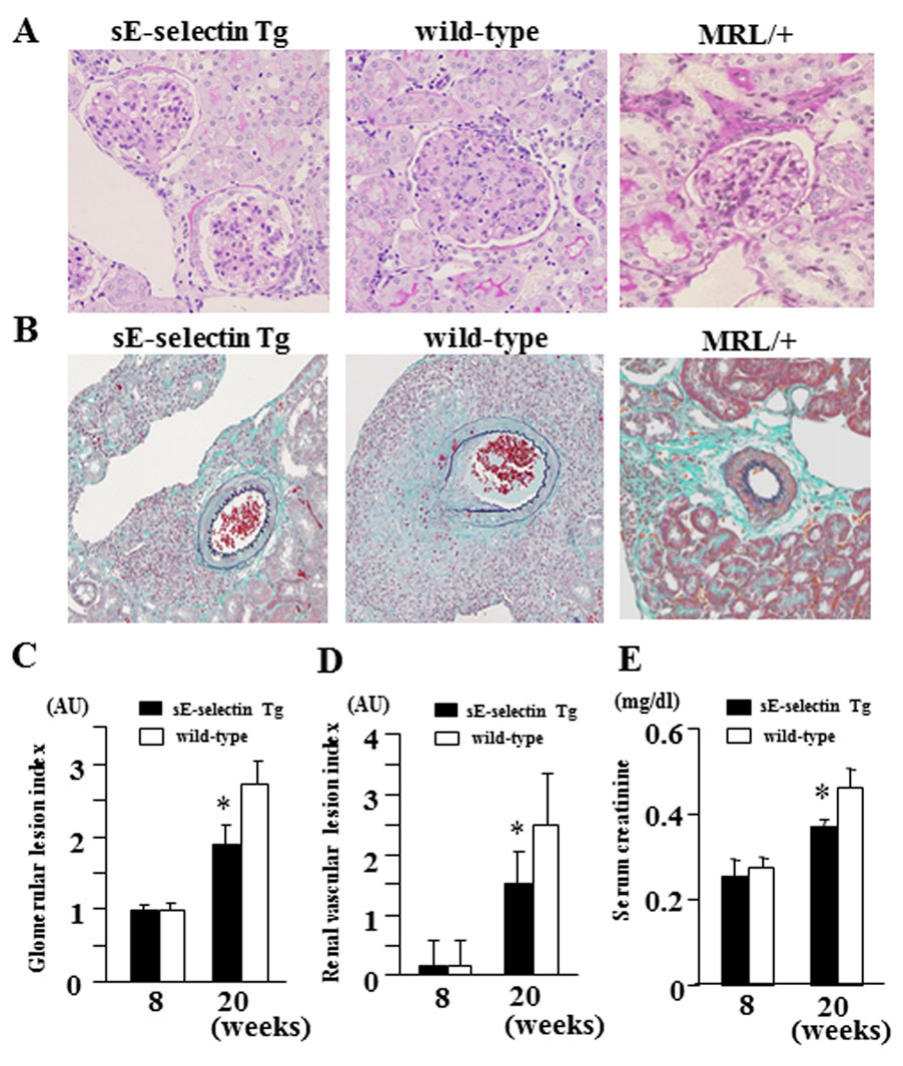
Circulating sE-selectin suppresses renal pathology in MRL/lpr mice. (A and B) Kidney sections from 20-week-old MRL/lpr and MRL/+ mice were stained with PAS (A) or Elastica-Masson (B). Representative PAS-stained sections showed that proliferative glomerular lesions were ameliorated in sE-selectin Tg mice, as compared to wild-type mice (A). Representative Elastica-Masson-stained sections showed that progression of vasculitis was suppressed in sE-selectin Tg mice, as compared with wild-type mice (B). MRL/+ mouse was as a control. (C–E) Quantitative analysis of renal injury in MRL/lpr mice. The glomerular lesion index (C), renal vascular lesion index (D), serum creatinine levels (E) were estimated as described in the Material and Methods. Data are expressed as means ± SD. *P < 0.05 versus wild-type MRL/lpr mice (*n* = 6 mice per group).

We then compared the extent of the renal damage in wild-type and sE-selectin Tg MRL/lpr mice. As shown in Figure 3A and B, 20-week-old wild-type MRL/lpr mice exhibited signs of progressive renal damage, including enlarged hypercellular glomeruli with elevated numbers of both resident cells and infiltrating leukocytes. In addition, within affected arteries, the vasculitis was characterised by marked accumulation of mononuclear cells with microgranuloma formation close to the external elastic lamina, destruction of the external elastic lamina, and intimal thickening. Notably, overexpression of sE-selectin in 20-week-old Tg MRL/lpr mice significantly ameliorated the glomerular hypercellularity and hyalinosis as well as the perivascular accumulation of mononuclear cells and destruction of vascular external elastic lamina, in comparison to wild-type MRL/lpr mice (Fig. 3A and B), but the renal damage in 20-week-old sE-selectin Tg MRL/lpr mice could be apparently detectable, in comparison to MRL/+ mice as a control (Fig. 3A and C).

To determine the histopathologic grade of the glomerular and vascular lesions, we calculated indexes reflecting the severity of the glomerular and vascular lesions for each mouse (see the Materials and Methods Section for details of the calculation). We found that the mean index for both the glomerular and vascular lesions was significantly lower in 16- and 20-week-old sE-selectin Tg MRL/lpr mice than in age-matched wild-type mice (*P* < 0.001; Fig. 3C and D). In addition, serum creatinine levels in sE-selectin Tg MRL/lpr mice were significantly lower than in wild-type mice (Fig. 3E). Immunofluorescent staining revealed the presence of glomerular E-selectin in sE-selectin Tg MRL/lpr mice, but the reactivity was lower than in wild-type mice (Fig. S3A). We therefore used real-time PCR to confirm that renal levels of E-selectin mRNA expression in sE-selectin Tg mice were significantly lower than in wild-type MRL/lpr mice (sE-selectin Tg: 1.53 ± 0.39 (AU), wild-type: 5.17 ± 1.01 (AU), *P* < 0.05). We also confirmed that renal E-selectin mRNA levels were significantly higher in sE-selectin Tg MRL/lpr mice than MRL/+ mice(sE-selectin Tg: 1.53 ± 0.39 (AU), MRL/+: 1.00 ± 0.24 (AU), *P* < 0.05). On the other hand, there was little or no difference in glomerular deposition of IgG3 between sE-selectin Tg and wild-type MRL/lpr mice (Fig. S3B).

### Accumulation of inflammatory cells in the kidneys of sE-selectin Tg and wild-type MRL/lpr mice

**Figure 4:**
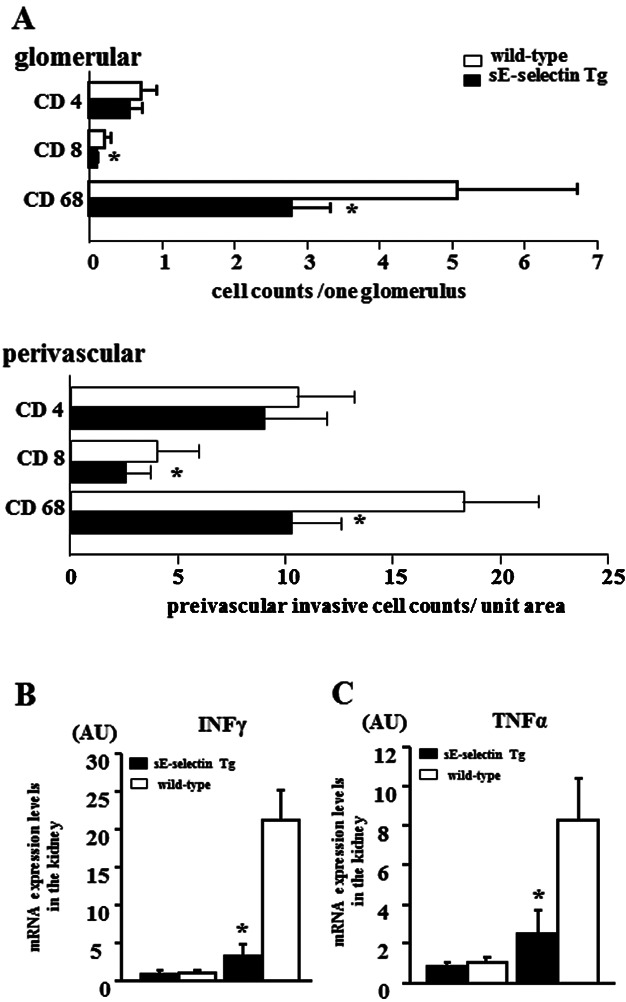
Quantitative analysis of glomerular and perivascular infiltration by mononuclear cells and renal expression of IFNγ and TNFα mRNA in wild-type and sE-selectin Tg MRL/lpr mice. (A) Numbers of CD4^+^ and CD8^+^ T cells and CD68^+^ macrophages infiltrating the glomerular and perivascular regions in 20-week-old MRL/lpr mice. (B and C) Quantitative analysis of IFNγ (B) and TNFα (C) mRNA expression in kidneys from 8- and 20-week-old MRL/lpr mice. Data are expressed as means ± SD. *P < 0.05 versus wild-type MRL/lpr mice (*n* = 6 mice per group).

Numbers of intraglomerular CD68^+^ macrophages and CD8^+^ T cells were significantly lower in 20-week-old sE-selectin MRL/lpr Tg mice than in wild-type mice (CD68: 2.79 ± 0.53 vs. 5.08 ± 1.66, CD8: 0.10 ± 0.02 vs. 0.22 ± 0.09, *P* < 0.05; Fig. 4A). In addition, infiltration by CD68^+^ macrophages and CD8^+^ T cells was markedly reduced in the perivascular region in sE-selectin Tg MRL/lpr mice, as compared to wild-type mice (CD68: 10.24 ± 2.35 vs. 18.25 ± 3.52, CD8: 2.54 ± 1.21 vs. 4.81 ± 1.98, *P* < 0.05; Fig. 4A). By contrast, although there were fewer CD4^+^ T cells infiltrating the glomerulus and perivascular region in sE-selectin Tg MRL/lpr mice than wild-type mice, the difference was not significant (glomeruli: 0.55 ± 0.19 vs. 0.71 ± 0.23, *P* = 0.21, vascular: 9.00 ± 2.95 vs. 10.52 ± 2.69, *P* = 0.37; Fig. 4A).

### Production of IFNγ and TNFα in the kidneys of sE-selectin Tg and wild-type MRL/lpr mice

IFNγ and TNFα reportedly play important roles in the progression of renal injury in MRL/lpr mice. For that reason, we assessed the expression IFNγ of and TNFα mRNA in total kidney prepared from 8- and 20-week-old sE-selectin Tg and wild-type MRL/lpr mice (Fig. 4B and C). In kidneys from 20-week-old sE-selectin Tg MRL/lpr mice, levels of IFNγ and TNFα mRNA expression were significantly lower than in kidneys from age-matched wild-type mice (TNFα: 2.52 ± 1.20 vs. 8.29 ± 2.10, IFNγ: 3.26 ± 1.62 vs. 21.3 ± 4.01, *P* < 0.01).

### Overexpression of sE-selectin prolongs survival

**Figure 5:**
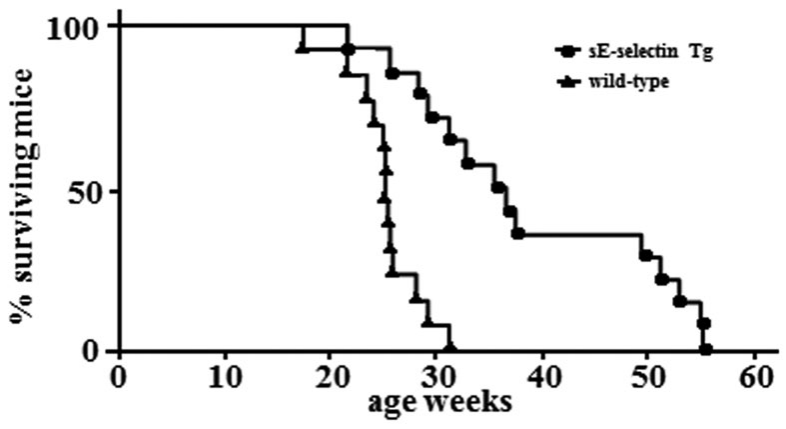
Kaplan–Meier analysis of survival of wild-type and sE-selectin Tg MRL/lpr mice. Survival analysis was based on the Kaplan–Meier method and the log Rank test (10 mice per group were followed for up to 55 weeks).

To determine whether overexpression of sE-selectin enhanced survival of MRL/lpr mice, we monitored sE-selectin Tg and wild-type MRL/lpr mice and recorded their deaths over time. Mouse survival was monitored for a period of 55 weeks, during which all of the mice died of renal failure. However, sE-selectin Tg MRL/lpr mice showed significantly longer survival than wild-type mice (*P* < 0.001; 50% survival: 26 weeks for wild-type MRL/lpr mice; 36 weeks for sE-selectin Tg mice; Fig. 5).

## Discussion

This is the first study to show that levels of E-selectin expression are significantly increased in lupus nephritis and vasculitis in MRL/lpr mice and that this effect is accompanied by increases in inflammatory cell infiltration. In addition, we showed that high levels of sE-selectin protein in serum can ameliorate the progression of lupus nephritis and vasculitis by suppressing inflammatory cell infiltration, especially infiltration by macrophages and CD8^+^ T cells.

The importance of adhesion molecules in the pathogenesis of lupus nephritis was suggested by the observation that expression of vascular cell-adhesion molecule-1 (VCAM-1) and intercellular adhesion molecule-1 (ICAM-1) are elevated in affected kidneys [20–25]. Moreover, several studies have shown that levels of soluble VCAM-1(sVCAM-1) and ICAM-1(sICAM-1) are also significantly increased in the serum and urine of patients with lupus nephritis and significantly correlate with disease activity indices [26–30]. These findings indicate that expression of ICAM-1 and VCAM-1 plays a critical role in the progression of lupus nephritis, and that sICAM-1 and sVCAM-1 can serve as markers of lupus nephritis activity. By contrast, elevation of sE-selectin in serum and urine is not reportedly associated with lupus nephritis activity; consequently, the importance of E-selectin and sE-selectin in the pathogenesis of lupus nephritis and vasculitis has remained unclear. It is well known that E-selectin is expressed on endothelial cells after activation by IL-1 or TNF-α [31,32], but unlike ICAM-1 and VCAM-1, it is not expressed on mesangial cells. In fact, we confirmed here that there is initially no detectable E-selectin expression along the capillary walls of glomeruli or small vessel walls. However, its expression was significantly increased during the progression of lupus nephritis and vasculitis and the accumulation of inflammatory cells in MRL/lpr mice, which suggests E-selectin plays an important role in the progression of lupus nephritis and vasculitis. It is now thought that sE-selectin is generated through the shedding of the membrane-bound E-selectin [33]. Membrane-bound E-selectin is present on the surface of cultured endothelial cells only transiently (for a period of 2–6 h), after which it is re-internalised [34]. We therefore speculate that sE-selectin levels may not be directly related to renal E-selectin expression levels and also may not be significantly associated with the activity of lupus nephritis.

To clarify the physiological importance of sE-selectin in lupus nephritis and vasculitis, we generated Tg mice that systemically overexpressed sE-selectin, and determined whether elevated sE-selectin in serum would modulate the incidence or progression of spontaneous lupus nephritis and vasculitis in MRL/lpr mice. Our initial hypothesis was that the overproduced sE-selectin would interfere with monocyte and/or lymphocyte infiltration of the affected kidneys through competitive inhibition of E-selectin binding of ligands such as E-selectin ligand-1 (ESL-1) and P-selectin glycoprotein ligand (PSGL-1), thereby inhibiting the development of lupus nephritis and vasculitis in Tg MRL/lpr mice. In addition, the extracellular portions of three selectins, L-selectin, P-selectin and E-selectin, share approximately 50% sequence homology. As previously reported, therefore, overproduction of sE-selectin could competitively block monocyte and/or lymphocyte infiltration mediated by L-selectin, as well as by E- and P-selectin [7,35,36]. Consistent with that idea, we found that sE-selectin suppressed the progression of both vasculitis and glomerulonephritis and ameliorated glomerular and perivascular inflammatory cell infiltration in the kidneys of MRL/lpr mice, which prolonged their survival. However, sE-selectin did not affect the pneumonitis, sialadenitis or arthritis also seen in MRL/lpr mice (data not shown).

It is well known that T cells express E-selectin ligands as well as L-selectin, and are important effector cells mediating glomerulonephritis and vasculitis in autoimmune disease [37–41]. In particular, CD8^+^ cytotoxic T cells (CD8^+^ Tc1 cells) and CD4^+^ Th1 cells are thought to contribute to the progression of lupus glomerulonephritis and vasculitis through release of inflammatory cytokines such as TNFα and INFγ [11,42–45]. Consistent with that idea, we observed significant CD4^+^ and CD8^+^ T cell infiltration of the glomeruli and perivascular regions in the kidneys of 20-week-old MRL/lpr mice. Similarly, in human proliferative lupus nephritis patients, we observed significant CD4^+^ and CD8^+^ T cell infiltration in the kidney, and found a significant correlation between renal E-selectin levels and renal infiltration by CD8^+^ T cells but not CD4^+^ T cells. Notably in that regard, we observed that renal invasion by CD8^+^ T cells was significantly ameliorated in 20-week-old sE-selectin Tg mice, but renal invasion by CD4^+^ T cells was not. In general, CD4^+^ Th1 cells express more functional E- and P-selectin ligand than CD4^+^ Th2 cells [46–48]. We therefore speculate that renal invasion by a CD4^+^Th1 subset among CD4^+^ T cells may be selectively ameliorated in sE-selectin Tg mice, although further studies will be needed to determine the specific association between E-selectin expression and renal migration of subsets of T cells in lupus nephritis. We also showed that renal expression of both IFNγ and TNFα mRNA was significantly lower in sE-selectin Tg MRL/lpr mice than wild-type mice, which likely reflects reductions in renal infiltration by CD8^+^ T and CD4^+^ Th1 cells.

On the other hand, there were no significant differences in serum ds-DNA titers, IgG3 levels or intraglomerular IgG3 deposition levels between sE-selectin Tg and wild-type MRL/lpr mice, in which IgG3 productions and depositions are regarded to play a critical role in the development of glomerulonephritis [49]. The ability of lymphocytes to selectively migrate to, or ‘home’ in on, a particular lymphoid organ is an important means of exposing lymphocytes to foreign antigens and stimulating expression of specific antibodies through interaction with antigen-presenting cells. Selectins, including E-selectin, play important roles in lymphocyte homing. Particularly important, however, are L-selectin, which is essential for lymphocyte homing, and the specialised endothelial cells that comprise the high endothelial venules (HEVs) within peripheral lymph nodes, which constitutively express functional ligands for L-selectin on their surface [50]. We therefore speculate that overproduced sE-selectin would have little effect on the interaction of L-selectin and its ligands on HEVs within lymph nodes, and that humoral immunity in MRL/lpr mice would be little affected by overproduced sE-selectin.

It is well recognised that renal involvement contributes substantively to morbidity in SLE patients. Numerous investigators have documented the serious prognostic implications of the diffuse proliferative glomerulonephritis accompanied by inflammatory cell infiltration seen in SLE patients [51–55]. For that reason strong immunosuppressive therapy is usually administered in these patients. At present, SLE treatment still depends on nonspecific immunosuppressive or immunomodulatory agents, such as nonsteroidal anti-inflammatory drugs, corticosteroids, cyclophosphamide and mycophenolate mofetil [1]. Despite, or because of, this armamentarium, several problems remain. All of the aforementioned drugs can have significant side effects and induce potent immunosuppression, which is often associated by serious infectious complications [1]. Therefore, identification of novel drugs that can suppress SLE, especially proliferative lupus nephritis, without major side effects would be highly desirable. In this study, we show for the first time that sE-selectin can ameliorate lupus proliferative nephritis and vasculitis without major immunosuppression, and that sE-selectin-mediated local suppression of inflammatory cell infiltration of affected kidneys can improve SLE prognosis. We therefore propose that sE-selectin has the potential to become an effective drug for the treatment of lupus proliferative nephritis and vasculitis. Moreover, its use in combination with immunosuppressive or immunomodulatory agents may enable the dosages of those agents to be reduced, thereby mitigating their side effects.

In conclusion, our findings show that sE-selectin inhibits the extravasation of inflammatory cells, especially macrophages and CD8^+^ cytotoxic T cells, by competitively inhibiting the interaction between endothelial E- and/or P-selectin and ligands expressed on the inflammatory cells. sE-selectin may therefore enable development of a new approach to the treatment of lupus nephritis and vasculitis whereby disease activity is suppressed without potent immunosuppression.

## SUPPORTING INFORMATION

**Figure S1.** Immunoperoxidase staining for CD68, CD8 and CD4 in glomeruli and small vessels in kidneys from 20-week-old MRL/lpr mice.

**Figure S2.** Immunoperoxidase staining for E-selectin, CD68, CD8 and CD4 in glomeruli from human proliferative lupus nephritis patient.

**Figure S3.** Circulating sE-selectin has no effect on the deposition of immunoglobulin or the expression of E-selectin in glomeruli from MRL/lpr mice. (A and B) Immunofluorescent staining with anti-E-selectin (A) and anti-IgG3 (B) antibodies.

**Figure S4.** RT-PCR analysis using total RNA from liver of both 20-week-old Tg and wild-type mice showed that the expression of sE-selectin was increased in Tg mice, as compared to wild-type mice. The following primers were used for detection of extracellular domin of E-selectin: 5′-TAC AAG TCC TCA TGT GCC-3′ (forward) and 5′-GGC TTC ACA GGT AGG CGG C-3′ (reverse) (product size: 290 bp).
