# Supplementary figures and images for: Enhanced expression of the soluble form of E-selectin attenuates progression of lupus nephritis and vasculitis in MRL/lpr mice
Source: Immun Inflamm Dis. 2013 Oct 30;1(1):37–46. doi: 10.1002/iid3.6 (PMC4217541; doi:10.1002/iid3.6)

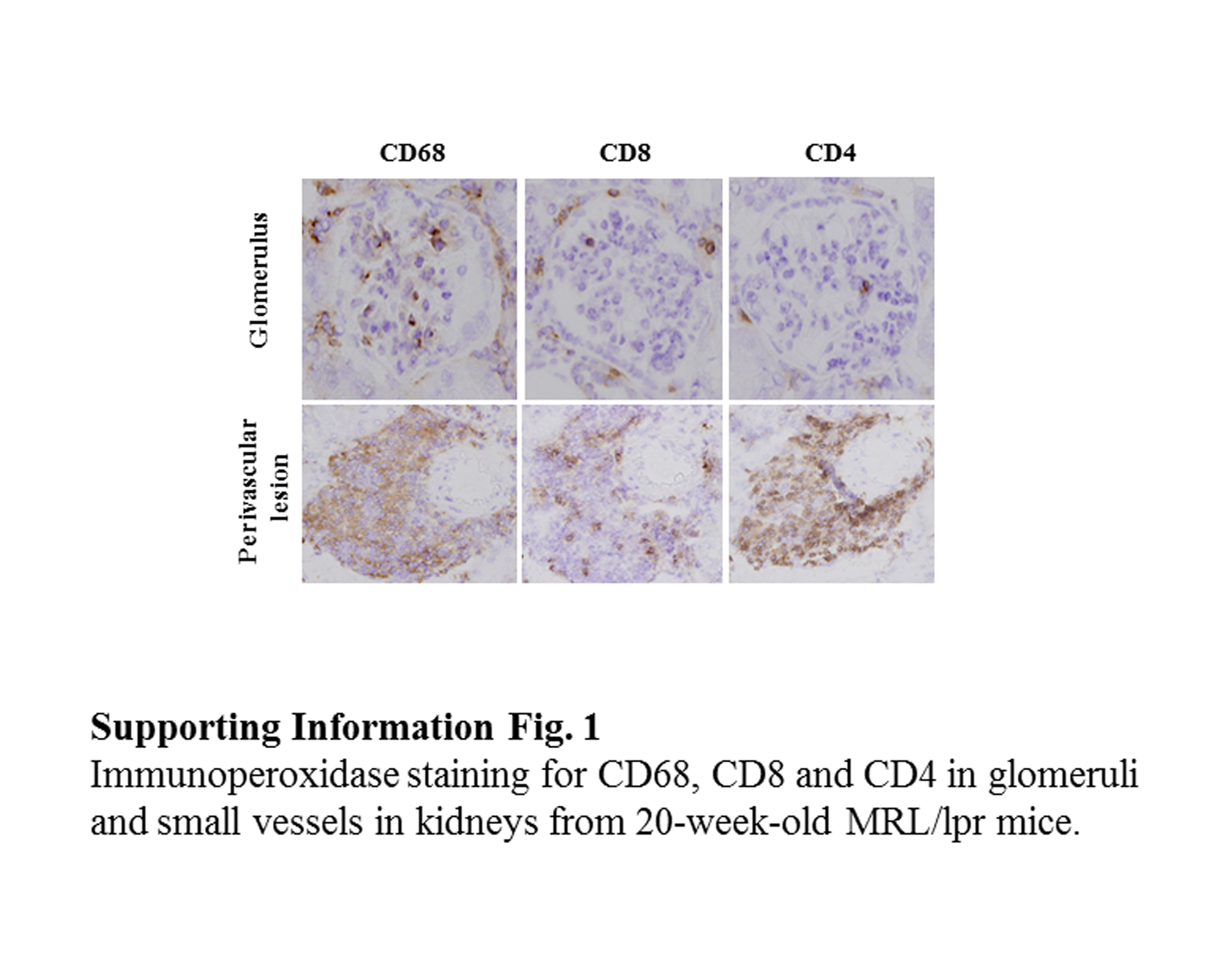

Supplement: Supplementary file 1 — Figure S1. Immunoperoxidase staining for CD68, CD8 and CD4 in glomeruli and small vessels in kidneys from 20-week-old MRL/lpr mice. [file iid30001-0037-SD1.tif]

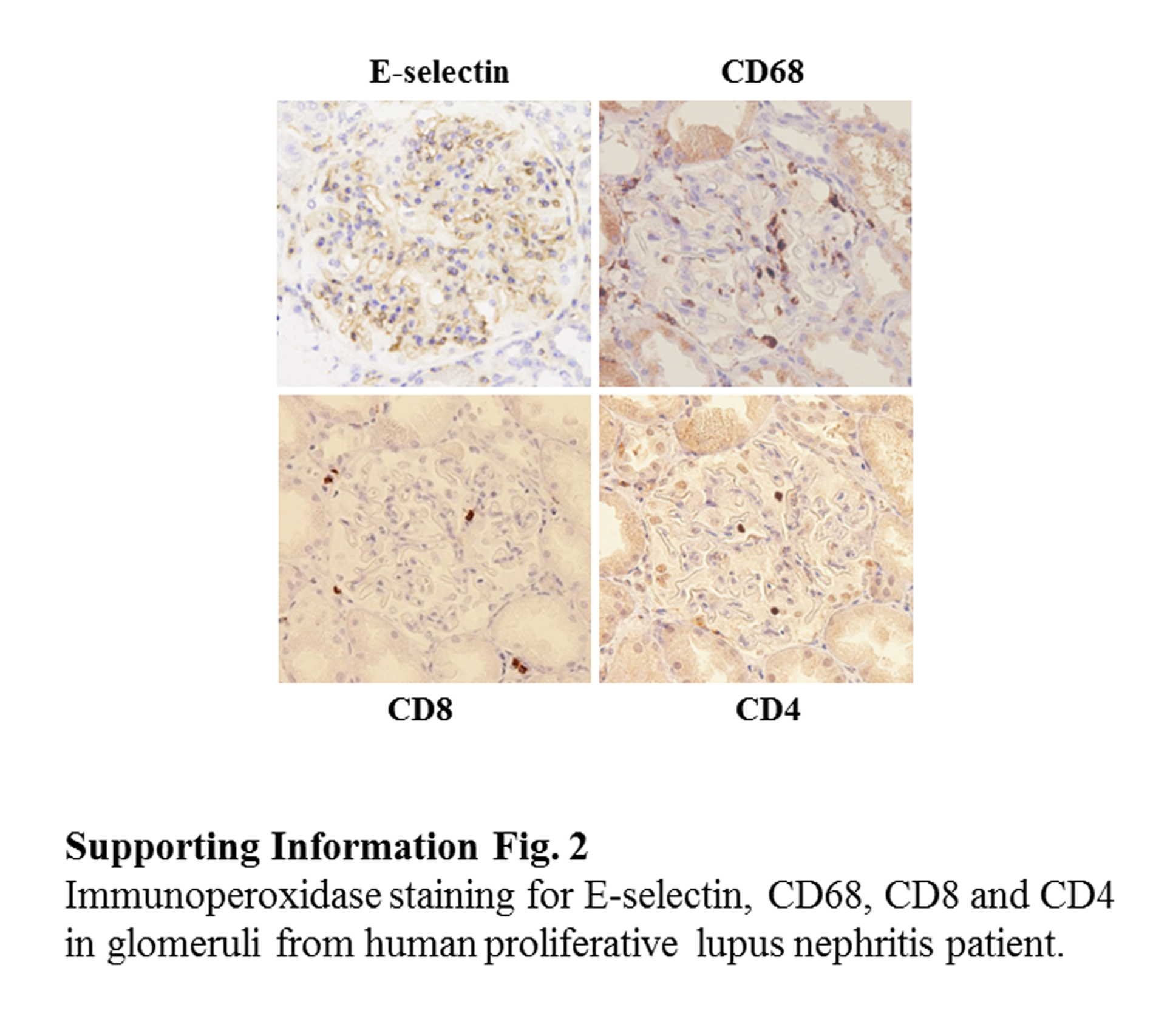

Supplement: Supplementary file 2 — Figure S2. Immunoperoxidase staining for E-selectin, CD68, CD8 and CD4 in glomeruli from human proliferative lupus nephritis patient. [file iid30001-0037-SD2.tif]

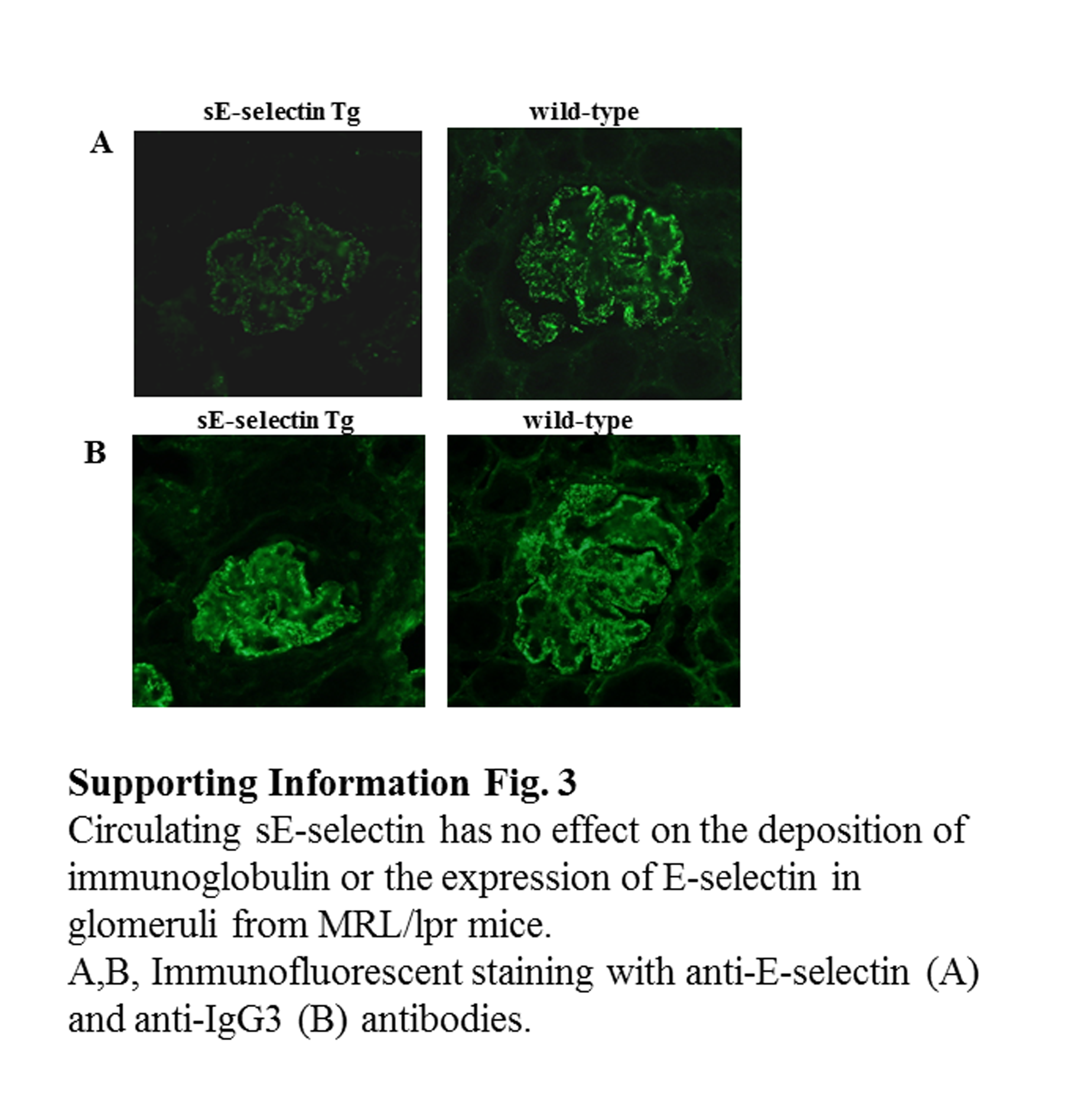

Supplement: Supplementary file 3 — Figure S3. Circulating sE-selectin has no effect on the deposition of immunoglobulin or the expression of E-selectin in glomeruli from MRL/lpr mice. (A and B) Immunofluorescent staining with anti-E-selectin (A) and anti-IgG3 (B) antibodies. [file iid30001-0037-SD3.tif]

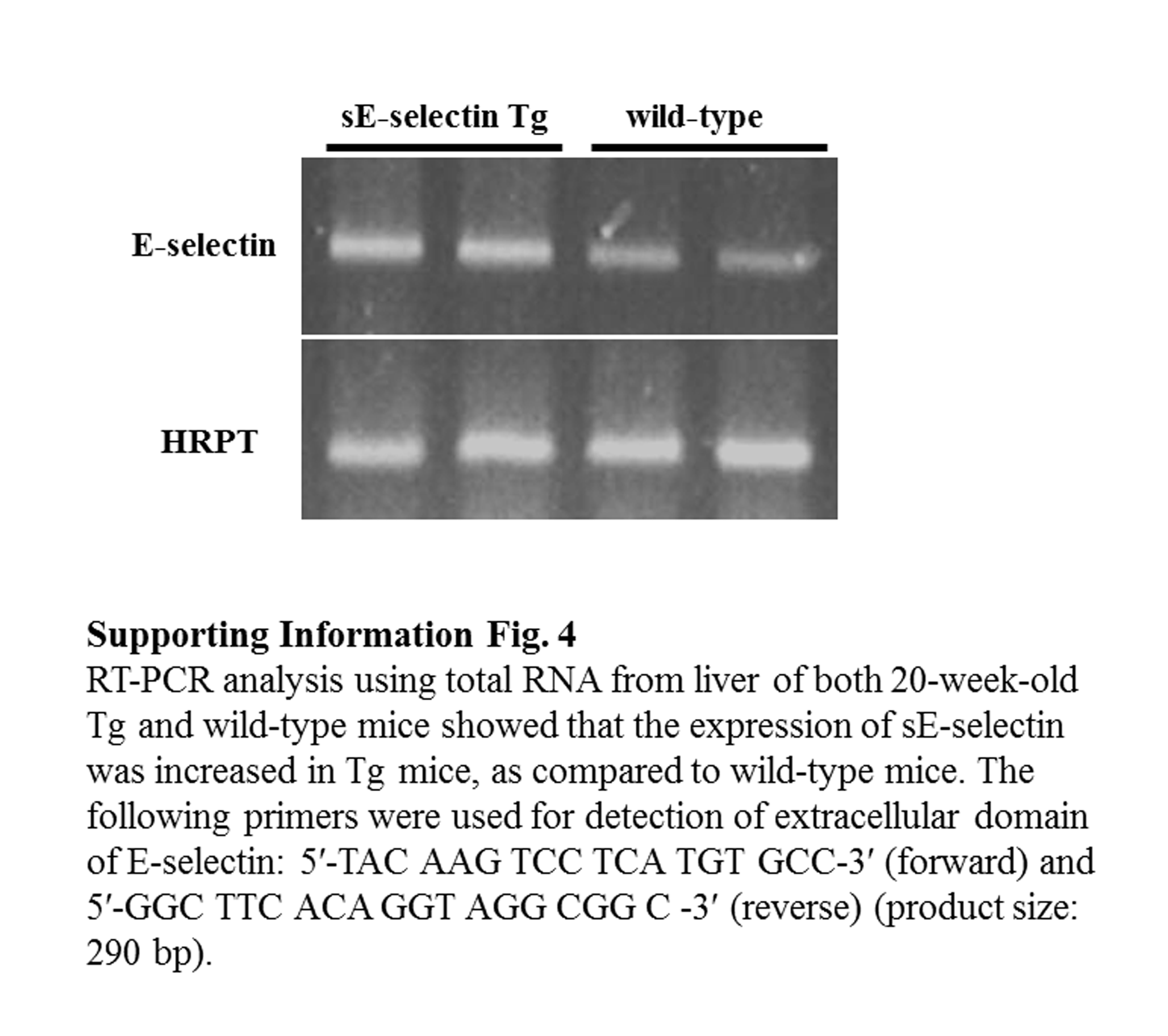

Supplement: Supplementary file 4 — Figure S4. RT-PCR analysis using total RNA from liver of both 20-week-old Tg and wild-type mice showed that the expression of sE-selectin was increased in Tg mice, as compared to wild-type mice. The following primers were used for detection of extracellular domin of E-selectin: 5′-TAC AAG TCC TCA TGT GCC-3′ (forward) and 5′-GGC TTC ACA GGT AGG CGG C-3′ (reverse) (product size: 290 bp). [file iid30001-0037-SD4.tif]
